# Plasma power effect on oxygen reduction reactivity of copper/nitrogen-doped graphene core-shell prepared via plasma solution

**DOI:** 10.1038/s41598-025-30697-9

**Published:** 2025-12-15

**Authors:** Amr Gangan, Ahmed M. Saeed, Alaa Fahmy

**Affiliations:** 1 Chemistry Department, Faculty of Science, Al-Azhar University, Cairo, Egypt; 2 Petrochemicals Department, Faculty of Engineering, Pharos University in Alexandria, Alexandria, Egypt

**Keywords:** Core-shell, Electrocatalyst, N-doped graphene, Oxygen reduction reaction, Liquid plasma, Chemistry, Materials science, Nanoscience and technology

## Abstract

Metal-carbon core-shell nanostructures have gained research interest, due to their valuable properties, such as high reactivity, stability, catalytic, optical, and electrochemical properties. However, the uses of metal-carbon core-shell nanostructures are limited due to the complicated synthesis processes. Therefore, developing a simple and fast method for synthesizing metal-carbon core-shell nanostructures is one of the main targets. In this work, the encapsulation of copper core by a nitrogen-doped graphene shell (Cu-NG) was successfully fabricated using a one-step solution plasma discharge process (SP) at different plasma powers. The structure of the prepared Cu-NG core-shell at different powers was confirmed and verified by X-ray photoelectron microscopy (XPS), high-resolution transmission electron microscopy (HR-TEM), X-ray diffraction (XRD), ultraviolet-visible spectroscopy (UV-Vis), Fourier transform infrared spectroscopy (FTIR), and Raman shift measurements. The structural analyses exhibited a fine core-shell structured nanoparticle with a size range of 5 to 15 nm, dependent on plasma discharge power. Notably, the N-doped graphene shell was favored at low power (120 W). The electrocatalytic activity toward the oxygen reduction reaction (ORR) of the obtained samples in an alkaline solution was acceptable. The effective ORR activity was possibly attributed to the synergistic effect of the copper core and N-doped graphene shell. This study provides eco-friendly and cheap alternative ORR catalysts with acceptable electrocatalytic activity.

## Introduction

 Fossil fuel is nonrenewable and releases many pollutants to the environment^1^, therefore, both academic and industrial fields are paying much attention to finding alternative sustainable energy resources^2–4^. Fuel cells have been widely studied as alternative energy sources. One of the most promising technologies has been identified as the proton exchange membrane fuel cell (PEMFC)^5–8^. It can convert chemical energy to electrical energy without causing pollution. A PEMFC typically uses hydrogen gas (H_2_) and oxygen gas (O_2_) as fuels. H_2_ molecules are oxidized at the anode and release electrons, which move through an external circuit to the cathode and reduce the oxygen molecules. To overcome the high activation energy of these reactions, different catalysts have been utilized to enhance the oxygen reduction reaction (ORR)^9^. The development of efficient and eco-friendly electrocatalysts for various energy conversion and storage applications has been a topic of great interest in the field of materials science and electrochemistry^10,11^. Platinum (Pt) is the most effective cathodic catalyst because it can promote the ORR at low temperatures^12^. Even though Pt-based catalysts have excellent ORR activity, the high cost is still one of the issues concerning and the biggest challenge. Recently, metal carbon support materials, which have high thermal and chemical stability and low cost, have been provided to the research and market^13^. Particularly, graphene is being widely used as a support for Pt and Pt-based catalysts. However, graphene contains only *sp*^*2*^ hybridized carbon–carbon bonding, which may change the surface chemistry of the catalysts and lower their catalytic activity^14^. Doping heteroatoms into the carbon structure of graphene enhances the ORR activity^15^. One promising approach is the utilization of metallic nanoparticles (NPs) encapsulated within N-doped graphene (N-G) matrices, as these hybrid structures can synergistically combine the unique properties of the individual components to enhance catalytic performance^16,17^. N-doped graphene with its high specific surface area, excellent electrical conductivity, and ability to stabilize metal NPs has been extensively explored as a support material for bimetallic electrocatalysts^18^. The incorporation of nitrogen in the lattice of graphene can further improve catalytic activity by modifying the electronic structure and providing additional anchoring sites for the metal NPs^19^. The synthesis of carbon-supported metal nanoparticles was fabricated via several methods, such as chemical vapor deposition and thermal treatment, and the laser-assisted synthesis method^20–22^. These methods are limited by high cost, complications, environmental issues, and multi-step operation. Solution plasma (SP) has emerged as a promising technique for one-step synthesis of N-G encapsulated NPs, as it allows for the simultaneous in-situ reduction of metal precursors and the nitrogen doping of graphene under mild conditions^10,23,24^. This facile, one-step approach eliminates the need for complex multi-step procedures, making it an attractive option for large-scale production. In this process, plasma is formed between two electrodes that are immersed in a solution. The molecules in the solution that are near to plasma region are activated and rapidly react with others to form products^25^. The structure of the obtained products depends on the type of solution and electrodes used in SP.

Therefore, the synthesis of N-doped graphene can be done in one step by using an organic liquid containing nitrogen, such as *N*,* N-dimethylformamide* (DMF). Phan et al.^12^. utilized the plasma (SP) method to prepare N-doped graphene encapsulated Pt-based bimetallic nanoparticles. They found acceptable reactivity toward the ORR in an acidic solution.

Previous studies focused on preparing metal-based N-doped graphene catalysts using different metals or solvents, with little attention given to plasma variables such as time, voltage, and current density. There are several factors that might affect prepared materials via plasma discharge in liquid or even in gas atmosphere such as electrodes material, power of plasma, gap distance between electrodes, duty cycle of plasma, pressure, and temperature^26^.

Jeong et al. described the creation of a transition metal electrocatalyst made of cobalt nanoparticles N-doped graphene nanoplatelets (Co@N/GNP) through plasma technique^27^. In this configuration, the cobalt nanoparticles served as active sites for the ORR, while the core-shell architecture with predominant pyridinic-N in the graphene contributed to exceptional catalytic efficiency, in an alkaline electrolyte achieving E_1/2_ = 0.87 V and E_onset_ = 0.98 V. Saadat and Kheradmand^28^ studied the performance of aluminum-air batteries by evaluating the impact of various electrocatalysts. The synthesized materials included N- and S-doped graphite (NSGr), N- and P-doped graphite (NPGr), graphene oxide (GO), N- and S-doped reduced graphene oxide (NSRGO), and N- and P-doped reduced graphene oxide (NPRGO), all used in conjunction with graphite. The electrocatalysts’ effectiveness for the ORR was analyzed through cyclic voltammetry (CV) and linear sweep voltammetry (LSV). For the NPRGO electrocatalyst in the discharge potential test, the initial value was recorded at 1.423 V, which decreased to 1.364 V after 600 s, reflecting a 3% reduction. The ORR catalytic activity of the cathodes was ranked from highest to lowest as follows: NPRGO, NSRGO, GO, NPGr, NSGr, and Gr.

In this work, Cu support-N-doped graphene (Cu-NG) was synthesized via a one-step synthesis of the SP method using a pair of copper metal electrodes in DMF. The synthesis was carried out at room temperature and atmospheric pressure at different plasma power. The obtained catalysts were characterized using X-ray photoelectron microscopy (XPS), high-resolution transmission electron microscope (HR-TEM), X-ray diffraction (XRD), UV–Visible, Fourier transform infrared (FTIR), and Raman spectroscopy to confirm the formation of Cu supported by N-doped graphene (Cu-NG). Their utilization as an ORR electrocatalyst for operating under an alkaline environment was also evaluated and discussed in detail.

##  Materials and methods

###  Materials

Nafions perfluorinated resin solution (5 wt%, Sigma-Aldrich), dimethylformamide (DMF, 99.5%) was used as media for plasma discharge, acetone (99.5%) and ethanol (absolute) were utilized for washing were purchased from El-Gomhouria, Cairo, Egypt. 1.0 mm diameter wires of copper were obtained from Elsewedy co., Egypt.

### Synthesis of Cu nanoparticles (NPs) encapsulated within N-doped graphene (N-G) matrices

**Fig. 1:**
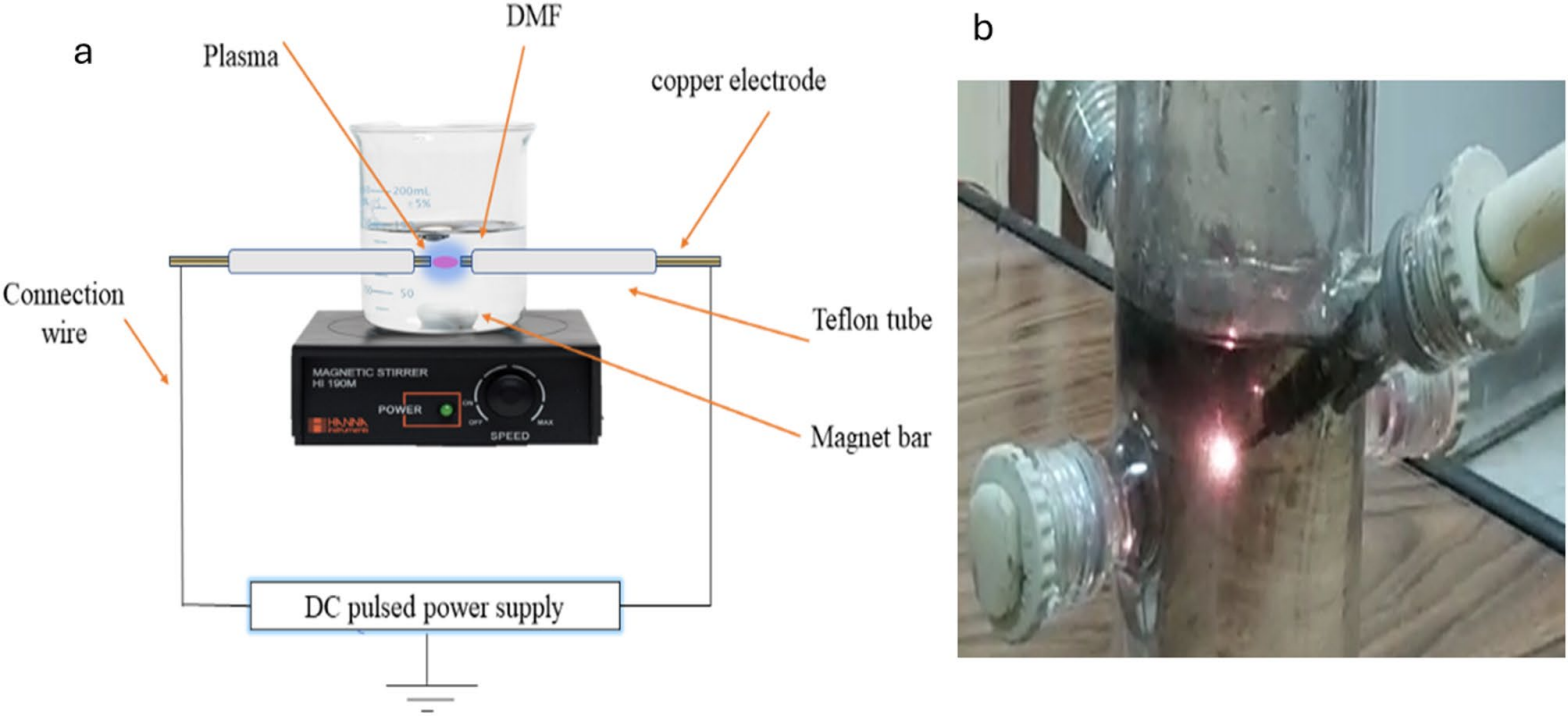
Schematic diagram showing the solution plasma (SP) process **(a)**, real image of SP-synthesis of Cu-NG **(b)**.

Employing two Cu wires to serve as an opposing electrode, Cu-coated with NG was produced via SP. The SP method involved plugging both electrodes into a glass reactor and insulating them using Teflon tubes. As illustrated in Fig. 1, the electrode tips were positioned in the middle of the reactor with just a 1.0 mm gap between them. The gap distance between the electrodes was optimized before the process (the best gap distance at which plasma ignition starts,1.0 mm). Between the two electrodes submerged in 100 mL of DMF, a handmade DC pulsed power supply was used to produce and sustain a plasma discharge. Furthermore, electrode erosion was minimized by using a short processing pulsed DC system (1.0 µs). Therefore, plasma was operated with a repetition frequency of 50 kHz and a pulse width of 1.0 µs. Continuous formation of black solid particles occurred during the plasma discharge. The experiments were conducted at room temperature, atmospheric pressure and different powers (120, 150 and 180 W). The minimum power required to initiate a discharge in the liquid depends on several factors, including pulse frequency and the liquid’s dielectric constant. Based on our experiments, plasma discharge in dimethylformamide (DMF) began at around 100 W; however, the discharge was unstable. Improved stability and sustained discharge were consistently observed at power levels above 110 W. While localized heating occurs in the plasma zone. Still, it was assumed to remain relatively stable due to the short duration and low total energy input. While the plasma itself may reach very high localized temperatures within its small discharge volume, the total energy delivered is low and the duration is short; thus, the energy dissipates quickly and the bulk solution does not exhibit a significant temperature rise^29,30^. The short duration of the plasma pulses, coupled with heat dissipation, will ensure that the liquid remains close to room temperature^31,32^. Following a 15-minute reaction, the particles were gathered, cleaned with acetone, and then dried for 12 h at 60 °C. The prepared catalyst was presented as Cu-NG-120, Cu-NG-150, and Cu-NG-180, respectively, after the preparation procedure was repeated at various plasma powers of 120, 150, and 180 W.

###  Instrumental analysis

The samples’ morphologies produced were examined using scanning transmission electron microscopy (STEM) in Jeol (JEM-2100 PLUS) equipped with Dry SD30GV Detector. X-ray diffraction (XRD PW 1,830 Diffractometer, Japan) in 2θ mode with Cu-Kα radiation (λ = 0.154 nm) at 40 KV and 40 mA was used to analyze crystal structures. The analysis of the Raman spectra was done utilizing a Witec alpha 300 R confocal Raman microscope (Raman, Witec, alpha 300R, Germany); A 532 nm laser was used to excite the samples at room temperature. A UV–visible spectrometer model (UV–vis, Jenway Model 6700) was used to record and analyze the materials’ optical characteristics and light absorption. The state of bonding between various components on the samples’ surface was characterized by X-ray photoelectron spectroscopy (XPS) (Thermo Fisher Scientific, USA). Al Kα radiation with an energy of 1487 eV was used for all measurements. This source’s power and emission voltage are set at 220 W and 11 kV, respectively. Throughout the study, the pressure in the chamber used for analysis was maintained at 10^− 7^ Pa, and Casa XPS was used to fit the spectra. With a sampling depth of 2.5 μm and an FTIR spectrometer model: Alpha 2 (Bruker), the chemical structure of the prepared samples was examined using the attenuated total reflectance (ATR) mode in the 400–4000 cm^− 1^ range.

### Tests involving electrochemistry

Cyclic voltammetry (CV), linear sweep voltammetry (LSVs) and electrochemical impedance spectroscopy (EIS) were used to test the resulting samples’ electrochemical characteristics. Three-electrode cells and an electrochemical working station (Potentiostat/Galvanostat/ZRA analyzer, GAMRY Reference 3000) were used to conduct electrochemical measurements. The three electrode cells were made up of an Ag/AgCl reference electrode, a 1 cm^2^ platinum plate counter electrode, and a rotating glassy carbon disk (GC RDE) of 3 mm diameter as the working electrode. At room temperature, the CV curves were recorded at a scan rate of 50 mV s^− 1^ in a 0.1 M KOH solution that was saturated with O_2_ and N_2_.

With a scan rate of 10 mV s^− 1^ and rotation speeds ranging from 300 to 1200 rpm, the LSV measurements were carried out on a GC RDE in an O_2_-saturated 0.1 M KOH solution.

In order to get a mirror-like surface, the GC electrode was rubbed on a polishing cloth using various alumina pastes and then ultrasonically cleaned before the measurements. 5 mg of the catalyst was dissolved in 490 µL of water, 490 µL of ethanol, and 20 µL of Nafions (5 wt%) to make the working electrode. After that, the ink of catalyst mixture was sonicated for 30 min to form a uniform suspension. The GC RDE was coated with 3 µL of the resultant catalyst suspension ink, which was then allowed to dry at room temperature in the air.

## Results and discussion

### FTIR analysis

**Fig. 2:**
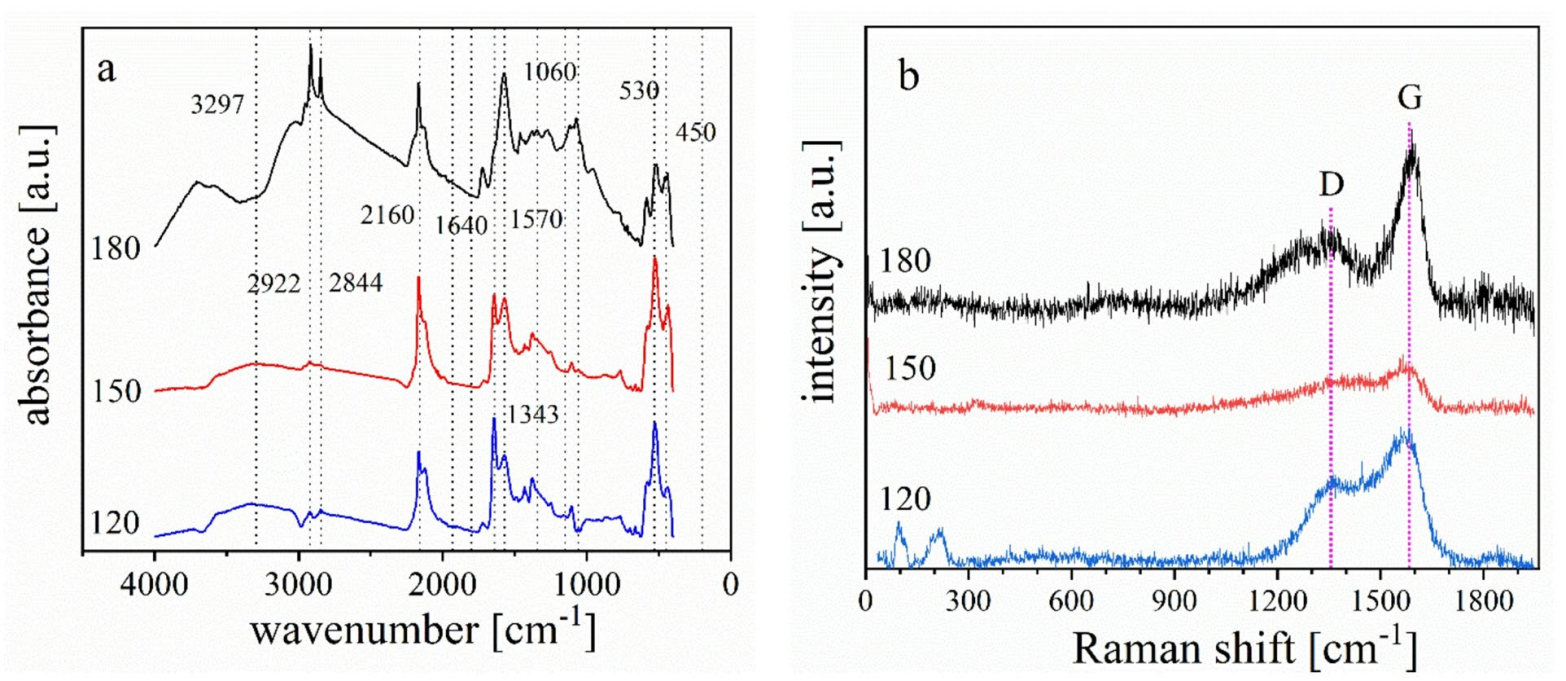
FT-IR spectra of Cu-NG catalyst at different powers (180, 150, and 120 W) pulsed plasma discharge (**a**). Raman spectra of Cu-NG catalyst with different powers of plasma (**b**).

The best analytical method for determining whether functional groups are present in NG is FT-IR spectroscopy. The typical FT-IR spectra of Cu-NG is depicted in Fig. 2a at different power. For the catalyst prepared at 180 W, an absorption peaks are obtained at ν 3400, 1032 and 1725 cm^− 1^ make it obvious that stretching of OH, C–O, and C = O, accordingly, are present. Additionally, it displays wide C–O stretching bands between *v* 1000 and 1400 cm^− 1^^33^. Higher levels of oxidation and the strong C–O epoxy group stretching (C–O–C) peak, which shows the simultaneous presence of O = C–OH (carboxyl), C–O–C (epoxy), and C–OH (hydroxyl) groups, are the causes of this stretching. This demonstrates that at high power, GO rather than NG forms. This observation was revealed by the appearing of the peak at 1342 cm^− 1^ in the catalyst prepared at 180 due to N–O stretching vibration present in N-GO^34,35^. At low plasma power, carbon atoms are replaced by nitrogen ones. This indicates by appearing peaks at ν 1640 and 1570 cm^− 1^ in NG at 150 and 120 W which represent, respectively, the sharp and powerful peaks of the C = C and C = N stretching bonds^36^. The Cu–O bond in the monoclinic crystal structure of CuO is responsible for the peaks at v 450 and 530 cm^− 1^^37^. In addition, the peak at 3297 cm^− 1^ is attributed to N-H stretching vibrations, which arise from nitrogen functionalities such as amine, pyrrolic, or pyridinic groups introduced during nitrogen doping of graphene. These groups are common in N-doped graphene and indicate successful nitrogen incorporation into the graphene lattice, often enhanced by plasma synthesis conditions^38,39^. Both peaks at ~ 2922 and 2844 cm^− 1^ are corresponding to C–H stretching vibrations. These are typically linked to the aliphatic C–H bonds in methyl and methylene groups, which may form at the edges of graphene sheets or on defect sites. The presence of these peaks suggests some surface functionalization, possibly resulting from plasma-induced reactions or residual organic species from precursor materials^39^. The relative intensities or positions of these peaks may change with different plasma powers (180, 150, 120 W), reflecting variations in dopant concentration and the extent of functionalization. Higher plasma power can result in enhanced doping and defect formation, leading to stronger or slightly shifted IR bands corresponding to the above functionalities. Generally, these assignments align with prevalent literature on N-doped graphene and are characteristic of successful nitrogen doping using plasma-based approaches.

### Raman analysis results

The produced Cu-NG samples’ Raman spectra at various discharge powers are shown in Fig. 2b. The two characteristic peaks in graphene-based materials are located at 1366 and 1588 cm^− 1^ and are known as the D and G bands. The G band is linked to the in-plane E_2g_ mode of single crystalline graphitic carbon atoms in the honeycomb lattice, while the D band indicates the presence of defects in the graphitic lattice with A_1g_ symmetry^40^. In general, the ID/IG ratio gives details on the disordered behavior of the graphene derivatives, in-plane and edge defects, and in-plane crystallite diameters. It is commonly known that adding N atoms to graphene can promote the production of many defects, leading to a high-intensity D band. This is because hceteroatom doping produces smaller nanocrystalline graphene domains^12,13^. It is evident from the D and G bands acquired for SP-prepared Cu-NG at varying power levels that there is no disorder at 120 and180 W, respectively. As demonstrated in Fig. 2b, the considerable increase in the D band 150 W clearly reveals the formation of high amount of defected N-doped graphene.

According to FTIR studies, the high power (180 W) may produce highly energetic ions and radicals, particularly OH radicals, which tend to produce GO instead of NG.

### XPS analysis

**Fig. 3:**
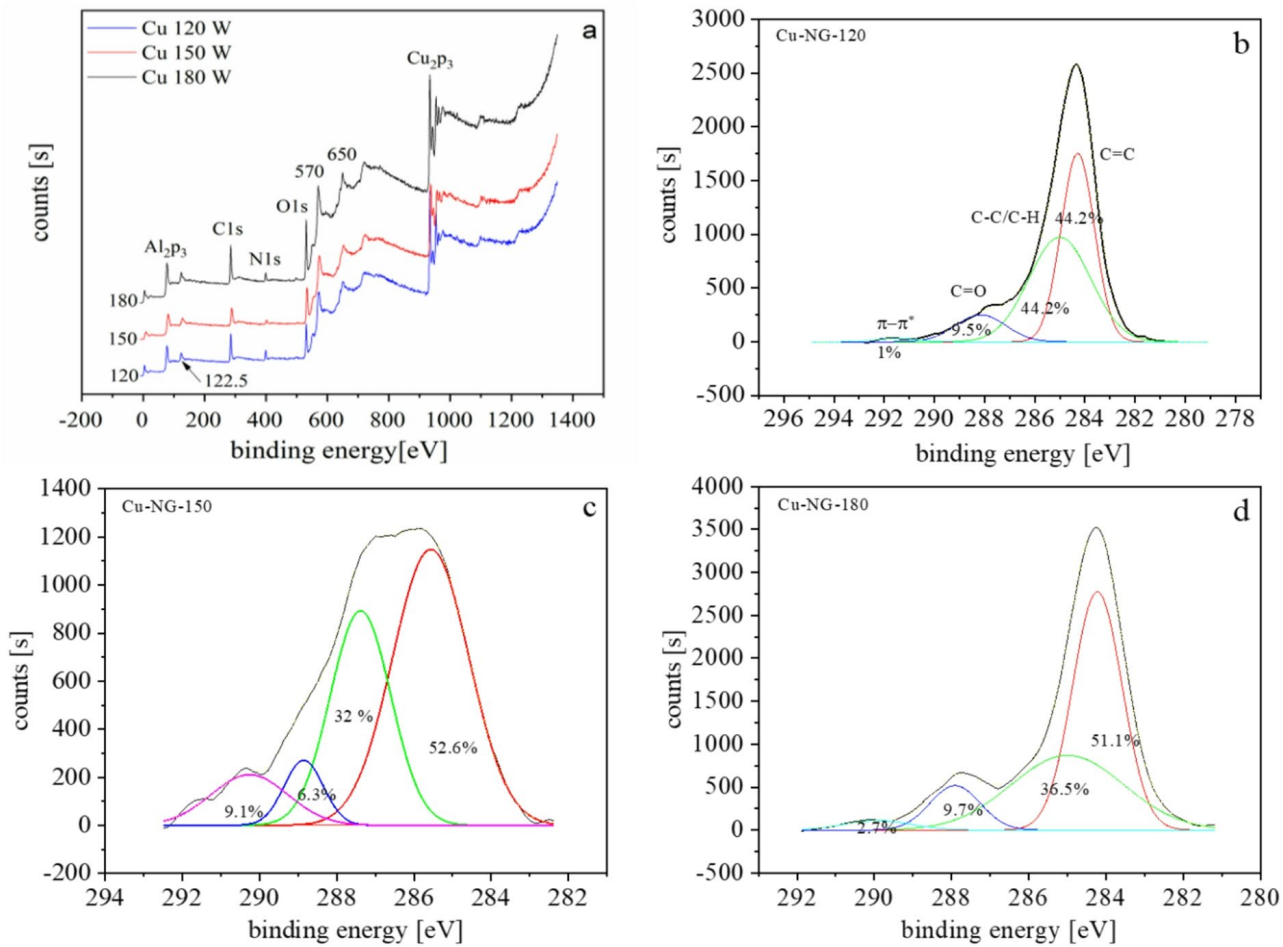
XPS survey scans of Cu-NG at different powers (**a**), the C1s peak and relative area under the peaks for Cu-NG at 120 W (**b**), 150 W (**c**), and 180 W (**d**).

The evidence of doping in Cu-NG by nitrogen is confirmed by XPS analyses. The dissociation and recombination of the molecules of DMF in the SP process may have produced nitrogen and oxygen as seen in Fig. 3a and their contents are depicted in Table 1. Additionally, the high-resolution XPS spectra was subjected to deconvolution to identify the elemental chemical states.

As seen in Figs. 3b, c, and d, four peaks were produced after deconvolution of the C1s spectra.

**Fig. 4:**
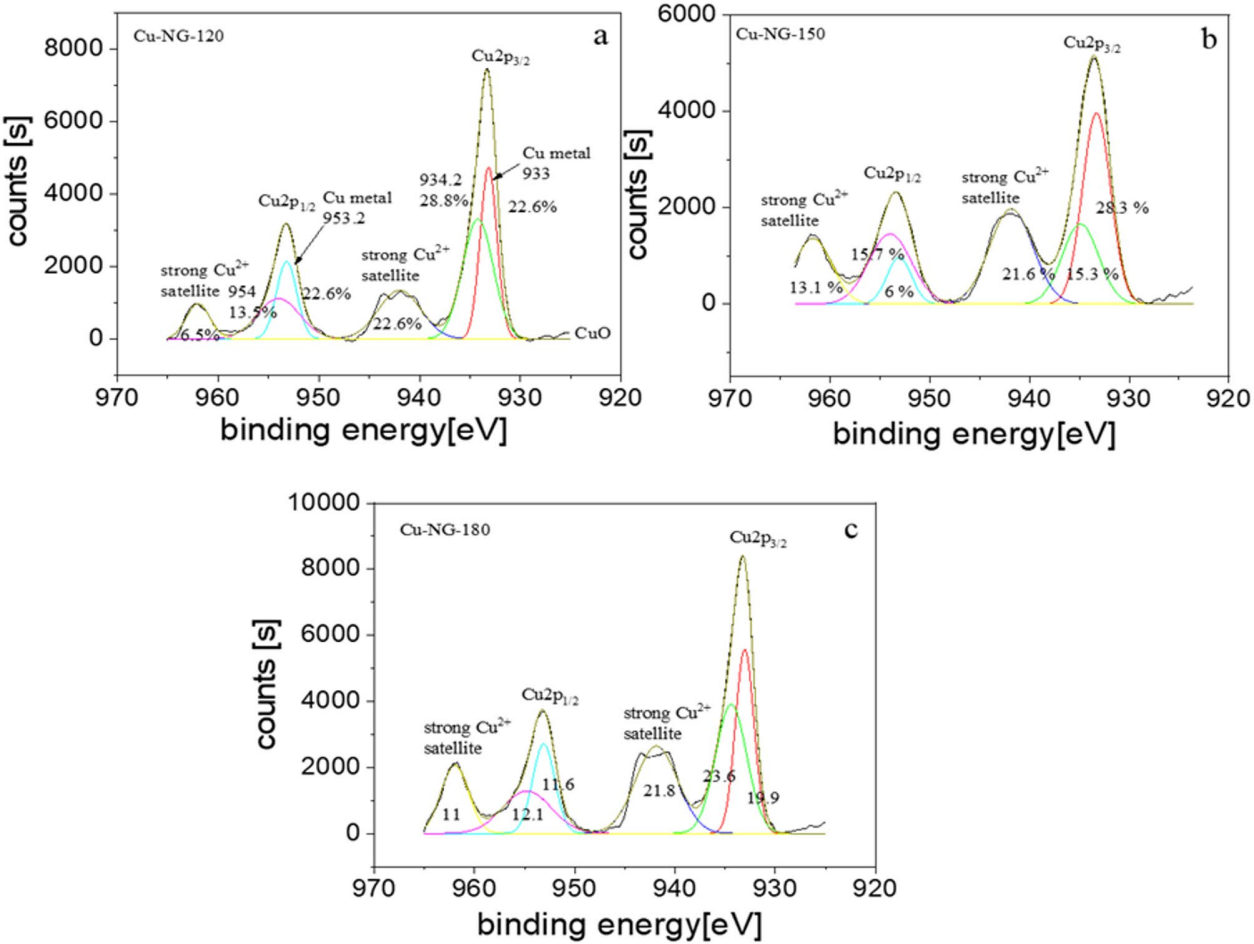
XPS spectra of the Cu2p peak and relative area under the peaks for CU-NG at different power: 120 (**a**), 150 (**b**), 180 W (**c**).

C = C of the carbon sp^2^ structures of NG is attributed to the strong peak at 284.2 eV^41^. Furthermore, C-H/C-C, C = N/C = O, and π-π* bonding occurrence peaks occur at ~ 285, 288.1, and 290.6 eV, respectively was detected, indicating the functionalization of the graphene by nitrogen and oxygen^42–45^. Cu 2p_3/2_ and Cu 2p_1/2_ were identified by deconvolution of high-resolution Cu 2p_3/2_ spectra into 933.06 and 953.1 eV peaks, respectively (Fig. 4)^46^.

Two prominent satellite peaks developed at 941.9 eV and 961.9 eV, respectively, confirming the presence of CuO. In addition, Cu (0) might be also observed at 933.06 eV (22.6%) and Cu(II) at 934.4 eV (28.8%) and 941.9 eV (22.6%); respectively^3,12^ indicating that oxidized Cu core (CuO) exists in Cu-NG which was in accordance with FT-IR results. The results reveal that the structure of Cu/GO at low power is more regular than expected. However, by studying Cu_2p_ peak, it was observed that CuO is the main component where the Cu metal is a trace, as shown in Figs. 4. Furthermore, Cu metal % is much higher in the sample that was prepared at 120 W than 150 and 180 W.

**Table 1:** Surface composition Cu-NG-120, as determined by XPS.

Peak	BE, eV	FWHM eV	Area (*P*) CPS.eV	Area %
Cu2p3	934.19	3.99	261397.2	57.10
O1s	531.23	4.18	77721.04	16.98
N1s	399.35	3.54	21333.5	04.66
C1s	285.35	3.83	66247.36	14.47
Al2p3	76.69	4.37	31123.32	06.79

**Fig. 5:**
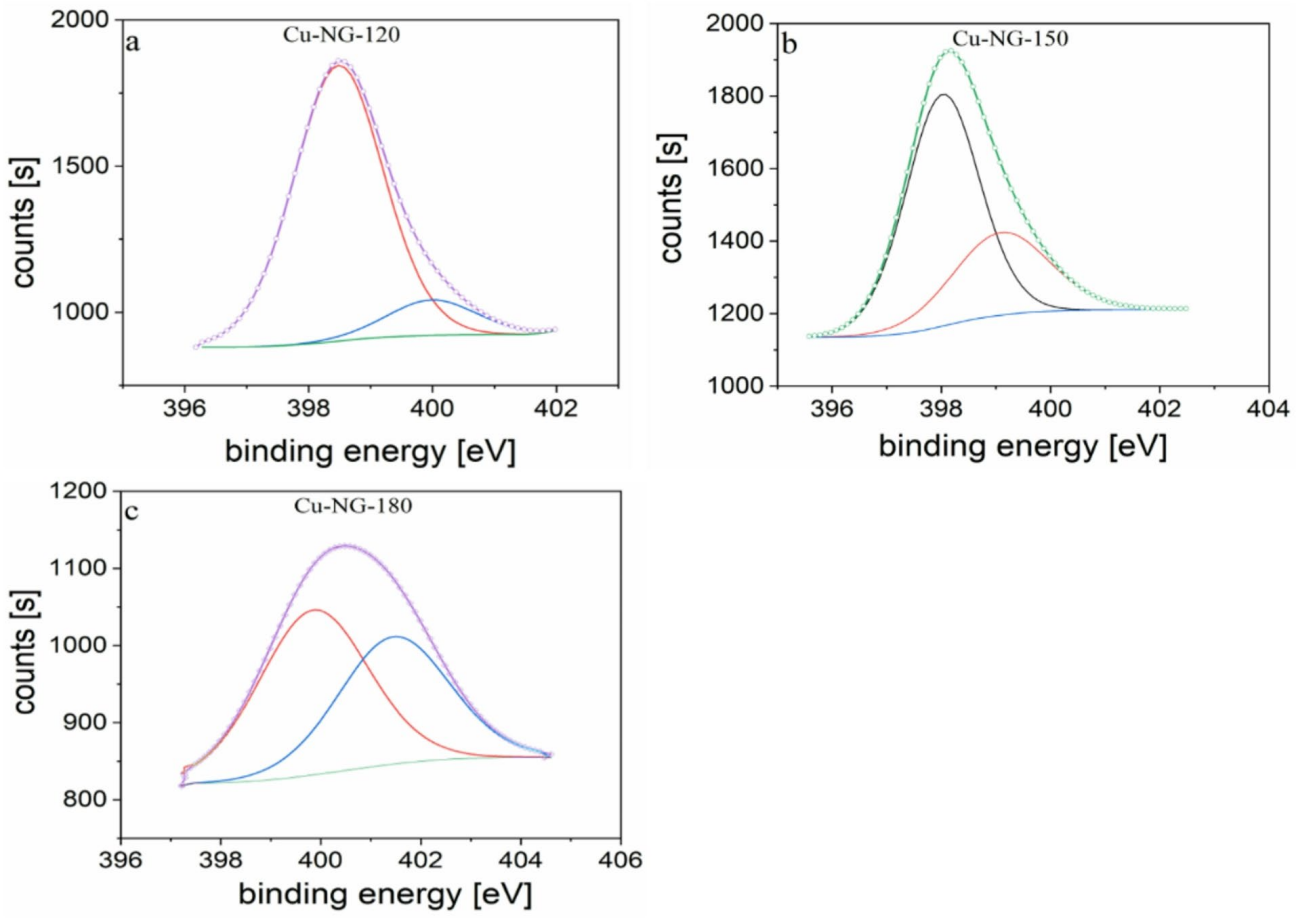
XPS spectra of the N1s peak for CU-NG at 120 W (**a**), 150 W (**b**), and 180 W (**c**).

Cu-NG-120, Cu-NG-150, and Cu-NG-180 high-resolution XPS N1s spectra are shown in Fig. 5. Three distinct components were used to match the spectra, which corresponded to various bonding states of N atoms, including graphitic-N (401.3 eV), pyridinic-N (398.4 eV), and pyrrolic-N (400 eV)^41,47^. Graphitic-N is a carbon atom substitute in the graphene structure, whereas pyridinic-N and pyrrolic-N are typically used to refer to the nitrogen atom near the edge of graphene frameworks. Each of pyridinic and pyrrolic N as the major species were detected in Cu-FG-120 and Cu-NG-150, while graphitic-N component was absent, while the graphic-N is found in Cu-NG-180. As plasma power was increased the probability of fragmentation process is increased causing formation of graphitic-N rather than pyridinic N. As reported in literature, pyridinic-N facilitates the adsorption of O_2_ and are essential for increasing the ORR activity in carbon materials doped with nitrogen^42,48,49^. Therefore, it was anticipated that Cu-NG-120 would have a greater beneficial impact on electrocatalytic activity than Cu-NG-180, which was subsequently verified by cyclic voltammetry studies.

### XRD analysis

**Fig. 6:**
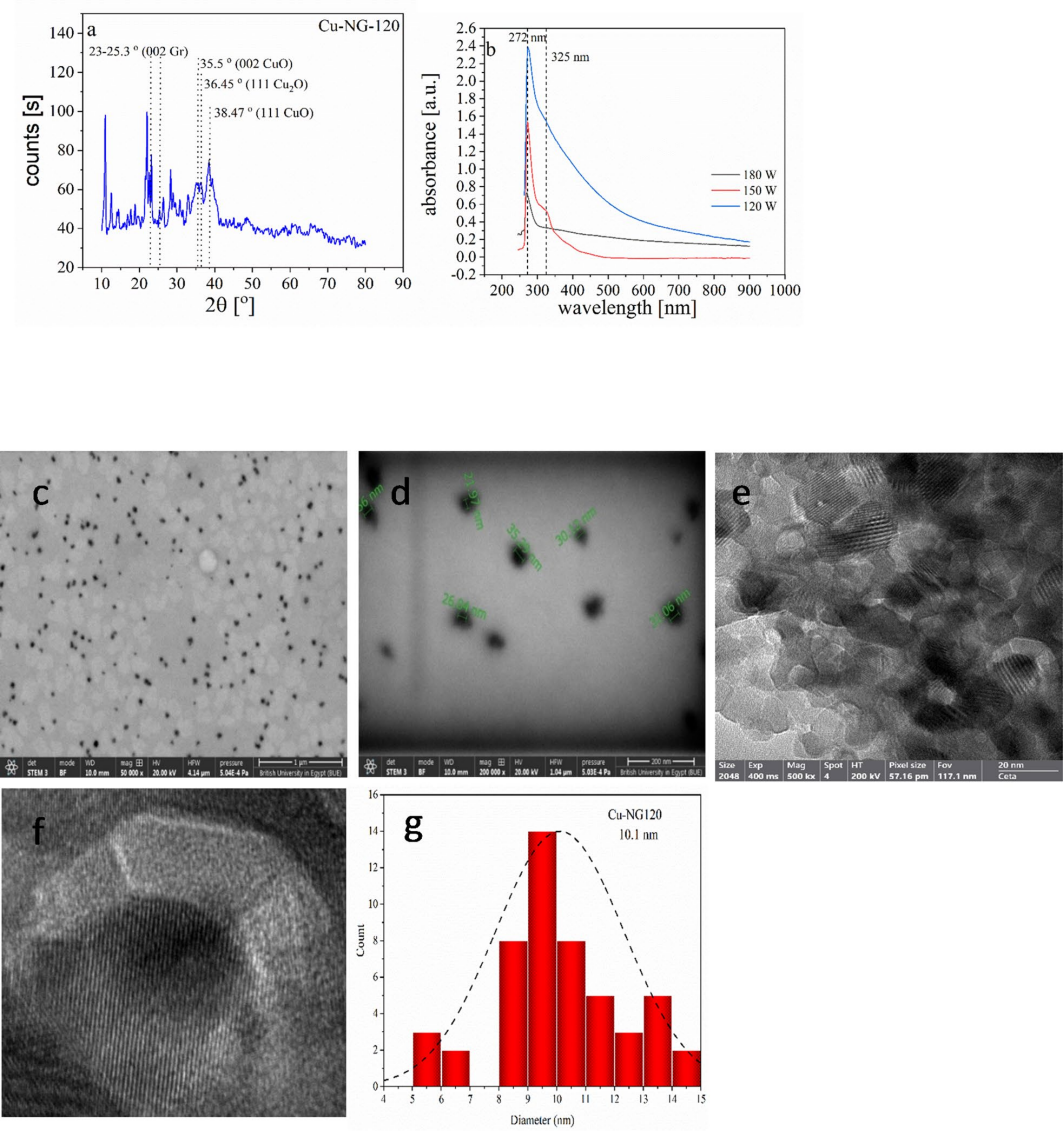
XRD patterns of CU-NG-120 as an example (**a**), UV–Vis. absorption spectra of Cu-NG catalyst at different power (180, 150 and 120 W) pulsed plasma discharge (**b**), TEM images of Cu-NG-120 (**c-e**), enlarged image of Cu-NG-120 (**f**), particles size distribution (**g**).

The crystal structure of the prepared catalyst was studied by using XRD. Figure 6a reveals the diffraction pattern of the Cu-NG-120 as an example, the peaks at 2θ = 35.55°, 38.47º, and 36.45º, corresponded to 002 and 111 planes of CuO, and 111 planes of Cu_2_O, respectively^50,51^. These oxide species were formed through the oxidation of Cu NPS during the plasma discharge process before encapsulation by the carbon shell. Moreover, the XRD patterns showed a diffraction peak at 2θ = 23.35º, 25.3º it was characteristic to the (002) plane of graphene^52^. In highly ordered graphite, the 002 reflection appears sharply around ~ 23– 26° 2θ due to the coherent stacking of graphene layers with a very low degree of turbostratic disorder^53,54^. The doping process usually results in a partial restoration of graphitic order with a peak closer to traditional graphite values^55^. The sharp XRD peaks may indicate the presence of residual graphitic ordering within the N-doped graphene matrix. Although nitrogen doping and oxidation–reduction processes are known to introduce a high density of defects that disrupt long-range ordering, these procedures may simultaneously induce local realignments or the formation of nanocrystalline regions that exhibit a high degree of layered stacking. For example, during thermal annealing in the presence of urea or ammonia, GO is reduced while nitrogen is incorporated into the carbon lattice, leading to the partial restoration of sp² hybridization and the re-formation of graphitic stacking^54,56^. Such local reorganization can result in domains with interlayer spacing approaching those of pristine graphite. The presence of a sharp and well-defined 002 peak in this region suggests that, at least in some portions of the material, there is not only a partial but an effective ordering that mimics the stacking found in high-quality graphite^57,58^. Moreover, the introduction of nitrogen and other defects may generally increase the D-band intensity (as illustrated in the Raman spectroscopy), the fact that a sharp 002 reflections are observed implies that, on a microscale, ordered graphitic domains can persist even in a highly defective environment^58^. Therefore, it is plausible that the observed sharp peaks reflect these preserved or recrystallized domains. In addition, it is known that few-layer graphene exhibits strong graphitic reflections, and nitrogen doping (N-doping) does not significantly alter these graphitic reflections. This is supported by studies showing that the intrinsic graphitic structure of few-layer graphene remains robust against N-doping, which mainly affects electronic properties without disrupting the characteristic graphitic lattice reflections^59^.

A further consideration arises in samples where copper is used as a substrate, leading to the formation of core–shell structures in which graphene layers encapsulate copper nanoparticles. In such cases, the underlying copper core can influence the overall XRD pattern. Copper, being a face-centered cubic (fcc) metal, exhibits its own set of diffraction peaks that, depending on their angular positions and relative intensities, may overlap with or modify the diffraction signals associated with the carbon shells^55,60^. Even if the primary copper reflections typically occur at positions different from 23–25°, interactions at the interface between the graphene shell and the copper core may induce strain in the carbon layers that shift or sharpen the graphitic reflections^57,61^. Therefore, when a copper core or copper residues are present, the sharpness of the XRD peaks observed in the 23–25° region may not exclusively reveal the intrinsic long-range order of the graphene layers. Instead, these peaks might be partially or predominantly influenced by the diffraction from or strain effects induced by the copper core.

The aforementioned possibilities are not mutually exclusive. In many practical systems, all these factors may be present simultaneously, leading to a complex XRD pattern where the sharp peaks around 23–25° could be the result of a weighted contribution from the mentioned reasons. As a result, complementary characterization techniques such as HRTEM, Raman spectroscopy, and XPS are essential to corroborate the existence of ordered domains^57,58^. As discussed in HR-TEM, the images (Fig. 6c and d) can reveal regions of well-aligned graphene layers coexisting with areas of significant curvature and disorder, while Raman spectroscopy (Fig. 2b) can differentiate between graphitic (G-band) and defect-induced (D-band) features, and XPS (Figs. 3 and 4, and 5, respectively) can provide insights into the bonding environment of the carbon and nitrogen atoms.

The encapsulation of the Cu core by the carbonaceous shell is proved by comparing the XRD patterns of Cu-NG-120 against that of fullerene. The diffraction patterns of Cu-NG-120 are observed at 2θ = 10.85°, 17.76°, 20.86°, 21.80°, 27.53°, 28.26°, 31.02°, 32.95°, which identify the (1 1 1), (2 2 0), (3 1 1), (2 2 2), (3 3 1), (4 2 0), (4 2 2), (5 1 1) planes, respectively. While the XRD peaks of fullerene based on literature appears at 2θ = 10.5°, 17.3°, 20.4°, 21.3°, 27.0°, 27.7°, 30.5°, and 32.4°^62–64^. However, the prepared Cu-NG-120 shows a slight peak shift toward the higher 2θ regions as compared to fullerene. This may be explained based on the fact of encapsulation of nitrogen-doped fullerene on the CuO nanoparticles that can change the lattice parameters of N-doped fullerene causing a lattice strain due to interaction between the core and the shell which shift in 2θ values^62^. Regarding the observation of fullerene peaks but not distinct graphene reflections, the interpretation of the peaks at 2θ = 10.85°, 17.76°, 20.86°, 21.80°, 27.53°, 28.26°, 31.02°, 32.95° as fullerene-like was based on previous literature^62^, which reported similar peaks for nitrogen-doped fullerenes. While few-layer graphene typically exhibits a broad (002) peak around 2θ = 25–26°, the solution plasma synthesis method, especially with nitrogen doping, can lead to the formation of highly disordered carbon structures, including fullerene-like cages or highly curved graphene fragments, rather than well-stacked graphene layers. This can result in a less pronounced characteristic graphene (002) peak, and instead, peaks associated with these more complex carbon structures may become dominant. The N-doping itself can indeed influence the stacking and crystallinity of graphene, leading to a more disordered structure, as highlighted by Geng et al. in their study^57^. They showed that N-doping can disrupt the graphitic order. Therefore, the absence of a sharp graphitic reflection and the presence of fullerene-like peaks might be indicative of the specific carbonaceous structure formed under our solution plasma conditions.

### UV results

Figure 6b presents the UV–Vis. absorption spectra of Cu-NG-120, Cu-NG-150, and Cu-NG-180. A characteristic absorbance of graphene (G) and graphene oxide (GO) in addition to Cu nanoparticles was observed. As literatures^65,66^, the GO demonstrates an absorption at 273 nm and 325 nm, which corresponds to the π → π* transitions of aromatic C–C bonds and the n → π* transitions of C = O bonds, respectively. Furthermore, a broad shoulder in the range from 425 to 550 nm might be related to the Cu nanoparticles of different sizes. It should be confirmed by TEM and XRD measurements. As a result, the shoulder from 300 to 550 nm is more pronounced with decreasing the power of plasma. It might be related to the amount of GO in the graphene shell.

###  Morphology of Cu-NG

The morphology of Cu-NG-120 was studied by HR-TEM and presented in Figs. 6c and d. The morphology exhibits agglomerated core-shell NPs having an average size of 5–15 nm Fig. 6e. The dark zone represented a Cu–-core while the grayish edges resemble to N-doped carbon–shell, respectively^67^. Figure 6d indicates that the shells contained a thick layer of shell, which coats the internal core. This coat stabilizes the metal core, which enhances the durability and catalytic performance of the prepared catalyst^67,68^.

### Electrochemical activity

**Fig. 7:**
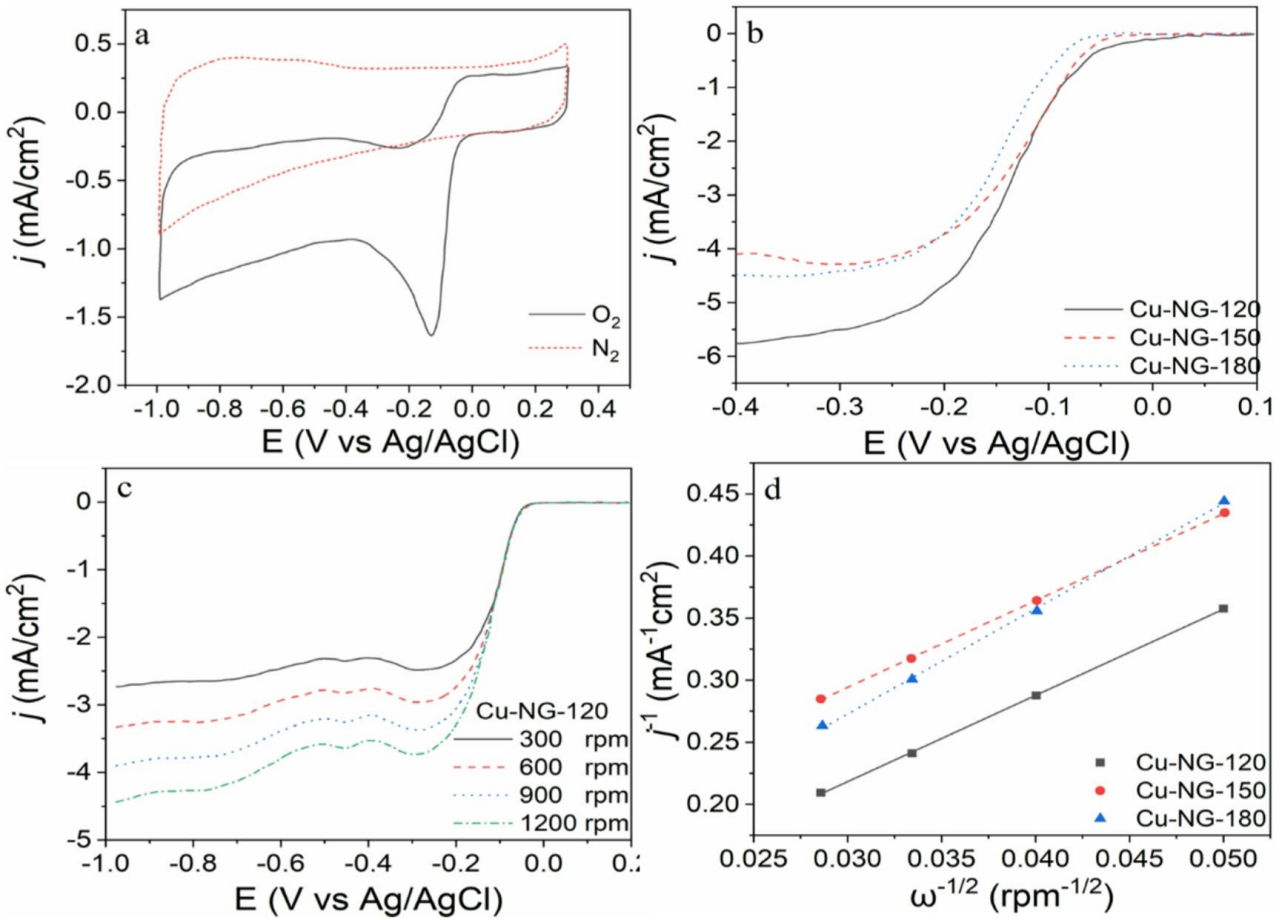
CV curves of Cu-NG-120 (**a**), LSV curves of Cu-NG-120, Cu-NG-150, and Cu-NG-180 at1200 rpm (**b**), LSV curves of Cu-NG-120 at 300–1200 rpm (**c**), and K–L plots at -0.4 V of potential resulted from Fig. 7a.

The Cu-NG-120 cyclic voltammetry (CV) curves are shown in Fig. 7a. There are no anodic or cathodic peaks in the potential range of 1.0 to 0.3 V in the N_2_-saturated electrolyte. However, the O_2_-saturated electrolyte’s CV curves show distinct cathodic peak potentials at 0.125 V, confirming Cu-NG-120’s electrochemical activity toward ORR^69^.

The LSV curves of Cu-NG-120, Cu-NG-150 and Cu-NG-180 recorded on an RDE were presented in Fig. 7b. For the ORR, the Cu-NG-180 curve has an onset potential of -0.085 V, which is more negative than the Cu-NG-150 curve’s (-0.064 V). On the other hand, Cu-NG-120 exhibits a substantially greater onset potential of -0.058 V. Additionally, in contrast to the other samples, which displayed a comparatively lower current density, the potential peak was shifted to a high current density. Therefore, compared to Cu-NG-150 and Cu-NG-180, the ORR activity of Cu-NG-120 was thought to have more beneficial impacts on electrocatalytic activity.

The LSV measurements on an RDE were carried out at various rotational speeds (300–1200 rpm) (Fig. 7c), to obtain more information about kinetics of the electron transfer for the ORR reactivity. From 300 to 1200 rpm, the limiting current densities of LSV curves increased progressively with increasing rotation rate, suggesting a shorter diffusion distance at higher rotation rates. Using the *Koutecky–Levich* (K–L) equation, the electron transfer number (n) per O_2_ molecule engaged in ORR was determined as follows^70^:

1 $$\:\frac{1}{J}=\:\frac{1}{{J}_{k}}+\frac{1}{{J}_{d}}=\frac{1}{{J}_{k}}+\frac{1}{B\sqrt{\omega\:}}$$

2 $$\:B=0.62\:nF{C}_{^\circ\:}{\left(D\right)}_{^\circ\:}^{2/3}({V)}^{-1/6}$$

where *J* is the measured limiting current density, *J*_*d*_ is the limiting diffusion current density, *J*_*k*_ is the limiting kinetic current density, *ω* is the electrode rotation (rad s^−1^), *F* is the Faraday constant (96485 C mol^−1^), DO is the oxygen diffusion coefficient, and C_O_ is the electrolyte’s bulk oxygen concentration. With n representing the total number of electrons transferred in oxygen reduction, and *V* is the kinematic viscosity of the electrolyte. For an oxygen-saturated 0.1 M KOH solution, C_O_ = *1.2 × 10*^*− 6*^ mol cm^−3^, D_O_ = *1.0 × 10*^*−5*^ cm^2^ s^−1^, and *V = 1.1 × 10*^*−2*^ cm^2^ s^−1^^12,70^. Based on the slope of the K–L plots, n value for Cu-NG-180, Cu-NG-150, and Cu-NG-120 at a potential of -0.4 V was determined and is shown in Fig. 7d. n values were calculated as 3.21, 3.4, and 3.9 for Cu-NG-180, Cu-NG -150, and Cu-N-120, respectively. These results suggest a higher selectivity of Cu-NG catalysts for four-electron pathway in ORR^3^.

Regarding the superior performance of Cu-NG-120 compared to Cu-NG-150 and Cu-NG-180, and the similar performance between Cu-NG-150 and Cu-NG-180, this may be explained based on the detailed characterization results presented in the manuscript as the following: 1. superiority of Cu-NG-120 as revealed by FTIR and Raman analysis, indicating the N-doped graphene shell was favored at low plasma power (120 W). Precisely, the FTIR showed prominent C = N stretching bonds at 120 W, and Raman analysis revealed a higher amount of defected N-doped graphene at 150 W but implied less disorder at 120 W compared to 150 W. More importantly, XPS analysis showed that the Cu metal percentage was significantly higher in the Cu-NG-120 sample compared to Cu-NG-150 and Cu-NG-180. The presence of more metallic Cu (Cu^0^) and a more favorable N-doping configuration (e.g., higher pyridinic-N content) at 120 W are crucial for enhanced ORR activity. Pyridinic-N is known to generate active sites for oxygen adsorption and reduction. The synergistic effect between the metallic copper core and the optimally N-doped graphene shell at 120 W likely contributes to its superior electrocatalytic performance, leading to a substantially superior onset potential and greater current density (Fig. 7b), such synergistic enhancement is reinforced by literatures indicating that pyridinic-N and metallic copper interactions improve ORR kinetics and catalytic efficiency^71,72^

**Fig. 8:**
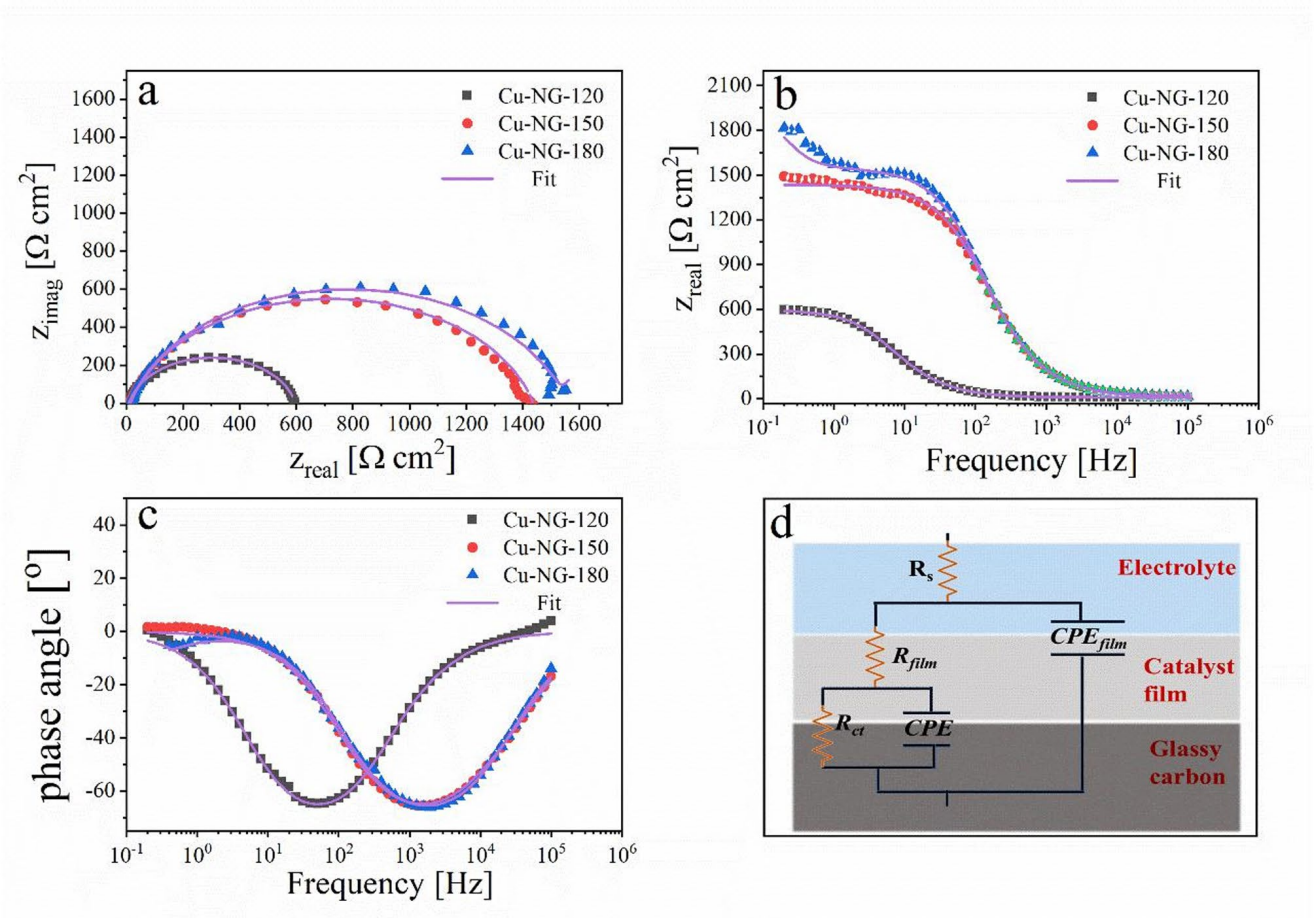
Electrochemical impedance spectroscopy (EIS) for prepared catalysts at 25 °C: Nyquist plot (a), Bode plots (b and c), and electrochemical equivalent circle used for fitting EIS data (d).

The electrochemical impedance spectroscopy (EIS) data shown in Figs. 8(a-c) provides important information about the electrode-electrolyte dynamics and interfacial charge transfer kinetics of the synthesized copper-nitrogen-doped graphene (Cu-NG) core-shell nanostructures, which were produced using solution plasma discharge at different power levels (120 W, 150 W, and 180 W). Together, the Nyquist plot (Fig. 8a), Bode magnitude (Fig. 8b), and phase angle (Fig. 8c) spectra show unique frequency-dependent characteristics that correspond to compositional and structural changes brought on by plasma power modulation^73^.

**Table 2:** EIS parameters for prepared catalysts in the aqueous solution of 0.1 M KOH at 25 °C.

	Rs (Ω cm^2^)	Rct (Ω cm^2^)	CPE		*R*_f_ (Ω cm^2^)	C_f_		Cdl (µF cm^− 2^)	Chi- square (χ^2^)
			Y_o_ (µ Ω ^−1^ s^*n*^ cm^− 2^)	*n*		Y_o_ (µ Ω ^−1^ s^*n*^ cm^− 2^)	*n*		
Cu-NG-180	14.59	1538	2.06	0.849	448.9	3.15	0.843	0.75	1.6 × 10^− 3^
Cu-NG-150	12.31	1147	3.15	0.832	278.6	163	0.929	1.01	6.4 × 10^− 4^
Cu-NG-120	12.57	575	32.00	0.843	12.63	56.5	0.889	15.21	6.8 × 10^− 5^

The impedance spectra were analyzed by fitting the experimental data to an equivalent circuit model composed of two-time constants as shown in Fig. 8d, and the different EIS parameters such as film resistance (R_f_), double-layer capacitance (*C*_dl_), charge transfer resistance (*R*_ct_) and constant phase element (Y_0_ and n) were collected in Table 2. The parameters in Table 2 were obtained by fitting the experimental EIS data using the fitting tools in Gamry Echem Analyst software. The consistency of the fits across all samples (as evidenced by the close agreement between experimental data and modeled curves) validates the proposed circuit model. Moreover, the fitted data indicate that the chi-square (χ^2^) values are around 10^− 4^. The lower value of χ^2^ indicates better agreement between the fitted data and the experimental results^74^.

Constant phase element (CPE) was used in order to deal with the non-ideal capacitance response. The impedance of the constant phase element (*Z*CPE) and the double-layer capacitance (*C*_dl_) are calculated by using Eqs. 3 and 4^73^:

3 $$\:Z\text{C}\text{P}\text{E}\:=[{{\:{Y}_{0\:}\left(\text{j}{\upomega\:}\right)}^{n}\:]}^{-1}$$

4 $$\:{C}_{\text{d}\text{l}}\:={\left({\:{Y}_{0}\:Rc\text{t}}^{1-n}\right)\:}^{1/n}$$

where *Y*_0_ = magnitude of the CPE, n = CPE exponent, ω = angular frequency and j = $\:{(-1)}^{1/2}$.

In the Nyquist representation (Fig. 8a), each sample exhibits a depressed semicircular arc in the high-to-medium frequency region, characteristic of a dominant charge transfer process at the catalyst-electrolyte interface. The diameter of this arc is proportional to R_ct_ which are 575 Ω cm^2^, 1147 cm^2^, and 1538 cm^2^ for Cu-NG-120, Cu-NG-150 and Cu-NG-180, respectively indicating progressively higher charge transfer resistance with increasing plasma power^75^. This trend aligns with the structural characterization obtained from XPS which clarify that the nitrogen-doped graphene shell is more effectively formed at lower plasma power (120 W), resulting in a thinner, more conductive, and defect-rich carbon matrix that facilitates electron transport between the copper core and the electrolyte. In contrast, higher plasma energies (150–180 W) may induce excessive graphitization or structural densification of the carbon shell, reduce active edge sites and increase interfacial resistance.

The Bode magnitude spectrum (Fig. 8b) supports this explanation, as the low-frequency region (where |Z| ≈ Rct) is lowest for Cu-NG-120, indicating higher charge transfer efficiency. Furthermore, the transition frequency, the point at which capacitive activity takes precedence over resistive behavior, changes toward higher frequencies for Cu-NG-120, implying faster interfacial kinetics and enhanced catalytic accessibility. The phase angle plot (Fig. 8c) confirms this, exhibiting a broader and deeper minimum for Cu-NG-120, indicating a more prominent capacitive response due to increased double-layer formation at the N-doped graphene surface. The presence of different nitrogenic species (pyridinic, pyrrolic, and quaternary N) in the graphene shell, as confirmed by XPS, contributes to higher pseudo capacitance and proton-coupled electron transport during the oxygen reduction reaction (ORR)^54^.

The fitted equivalent circuit parameters ( Table 2) show that the Cu-NG-120 exhibit the lowest R_ct_ and highest CPE values, reflecting greater electroactive surface area and favorable electron transfer kinetics. The R_f_ component, associated with the resistance of the carbon shell itself, which also minimized in Cu-NG-120 due to optimal nitrogen doping and reduced shell thickness, thereby minimizing ohmic losses. Overall, these EIS data give direct electrochemical evidence to support the structural assertion that Cu-NG ORR activity is mediated by a synergistic interaction between the metallic copper core and the N-doped graphene shell. The core promotes electron conduction, whereas shell controls adsorption energies and stabilizes reactive intermediates. The observed performance hierarchy Cu-NG-120 > Cu-NG-150 > Cu-NG-180 indicates that plasma power is an important synthetic parameter for modifying both morphology and electrochemical performance.

## Conclusions

This work presents an efficient one pot mothed using only DMF as the reaction solution at room temperature and atmospheric pressure to prepare Cu core encapsulated by N-doped graphene -shell as efficient ORR catalysts. N-Doped graphene acts as the protective shell, which enhances the reactivity, stability, and durability of the catalyst. Moreover, the structure of N-doped shells can be tuned by varying plasma discharge power to achieve the appropriate ORR reactivity. By decreasing plasma power, N-doped shell has greater content of pyridinic-N over graphitic-N, which is favorable for ORR process. In addition, the incorporation of N atoms in the graphene shell could cooperate with copper core and raise the selectivity of catalyst toward four-electron mechanism for ORR, which is favored for fuel cell applications. In general, it was found that the solution plasma showed a great ability as a promising, economic, and eco-friendly tool for development of a new high-performance catalyst, as an alternative to commercial Pt/C catalyst in fuel cell applications.

## Data Availability

All raw data of measurements is available and could be shared when requested, both corresponding authors (Alaa Fahmy) are fully responsible for providing all data requested.
